# *CD226* rs763361 Is Associated with the Susceptibility to Type 1 Diabetes and Greater Frequency of GAD65 Autoantibody in a Brazilian Cohort

**DOI:** 10.1155/2014/694948

**Published:** 2014-05-07

**Authors:** Teresa Cristina Colvara Mattana, Aritania Sousa Santos, Rosa Tsuneshiro Fukui, Debora Teixeira Oliveira Mainardi-Novo, Vinícius Silva Costa, Rosa Ferreira Santos, Sergio Russo Matioli, Maria Elizabeth Rossi da Silva

**Affiliations:** ^1^Laboratório de Carboidratos e Radioimunoensaio (LIM 18), Hospital das Clínicas da Faculdade de Medicina da Universidade de São Paulo, Avenida Dr. Arnaldo 455, 01246-903 São Paulo, SP, Brazil; ^2^Departamento de Genética e Biologia Evolutiva, Instituto de Biociências da Universidade de São Paulo, Rua do Matão 277, 05422-970 São Paulo, SP, Brazil

## Abstract

*CD226* rs763361 variant increases susceptibility to type 1 diabetes (T1D) in Caucasians. There is no data about *CD226* variants in the very heterogeneous Brazilian population bearing a wide degree of admixture. We investigated its association with T1D susceptibility, clinical phenotypes, and autoimmune manifestations (islet and extrapancreatic autoantibodies). *Casuistry*. 532 T1D patients and 594 controls in a case-control study. Initially, *CD226* coding regions and boundaries were sequenced in a subset of 106 T1D patients and 102 controls. In a second step, two *CD226* variants, rs763361 (exon 7) and rs727088 (3′ UTR region), involved with *CD226* regulation, were genotyped in the entire cohort. C-peptide and autoantibody levels were determined. No new polymorphic variant was found. The variants rs763361 and rs727088 were in strong linkage disequilibrium. The TT genotype of rs763361 was associated with TID risk (OR = 1.503;  95%  CI = 1.135–1.991; *P* = 0.0044), mainly in females (*P* = 0.0012), greater frequency of anti-GAD autoantibody (31.9% × 24.5%; OR = 1.57; CI = 1.136–2.194; *P* = 0.0081), and lower C-peptide levels when compared to those with TC + CC genotypes (0.41 ± 0.30 ng/dL versus 0.70 ± 0.53 ng/dL *P* = 0.0218). *Conclusions*. The rs763361 variant of *CD226* gene (TT genotype) was associated with susceptibility to T1D and with the degree of aggressiveness of the disease in T1D patients from Brazil. Ancestry had no effect.

## 1. Introduction

Type 1 diabetes is a heterogeneous autoimmune disorder characterized by severe pancreatic *β*-cell loss [1–4]. Discoveries of genetic variants associated with risk to T1D have provided new insights into their functional outcomes and affected pathways and until now more than 60 loci across the human genome have been associated with T1D susceptibility (http://www.t1dbase.org). The major histocompatibility complex (MHC) contributes to the main risk, followed by the insulin and the protein tyrosine phosphatase nonreceptor 22 (PTPN22) genes [2-4], including in the Brazilian population [5–7]. Nevertheless, established genetic associations explain only half of the genetic risk of T1D, indicating that other loci may concur with it.

Genetic and environmental factors contribute to the different incidence of T1D among populations. Indeed, genetic risk differ among different populations and sometimes even among groups with the same ethnicity [2, 3]. The Brazilian population is one of the most heterogeneous in the world with a wide degree and diverse patterns of admixture. Major contributions come from European ancestry (0.771), followed by African (0.143) and Amerindian ancestries (0.085) [8]. Although the incidence of T1D seems to be increasing in Brazil, scarce information is available about genetic and epidemiological data in this country [9].

Genome-wide association studies (GWAS) identified the association between the nonsynonymous p.Gly307Ser coding variant (rs763361) in* CD226* gene, located at 18q22.3 and T1D susceptibility in a European population [10]. The* CD226* gene encodes a 67 kDa glycoprotein (336 amino acids) also known as DNAX accessory molecule-1 (DNAM-1).* CD226* is constitutively expressed in the majority of CD4+ and CD8+ T lymphocytes, natural killer (NK) cells, monocytes/macrophages, platelets and megakaryocytes, and a subset of B lymphocytes, most of which are involved in immune mechanisms leading to *β*-cell destruction [11]. The poliovirus receptor (CD155) and its family member, nectin 2 (CD112), were identified as the ligands for CD226 [12] and interaction of CD226 with the ligands inducing NK cell and CD8+ T cell-mediated cytotoxicity and cytokine secretion [13].

Recently, Lozano et al. [14] verified that CD226 and its ligand CD155 were decreased on Th2 polarized naïve T cells, whereas both were highly expressed under Th17 conditions. CD226 provides a positive costimulatory signal for T cell proliferation and enhances proinflammatory cytokine production, thereby shifting the balance between Th1/Th17 and Th2 cells toward a proinflammatory response. Blockade of CD226 signaling decreased INF-gamma and IL-17 production. These data suggested that CD226 blockade may represent an alternative approach for treatment of Th1- and Th17-driven autoimmune diseases, allowing inhibition of T cell proliferation and proinflammatory cytokine production while sparing Th2 response.

Further, a new axis related to human T cell function, TIGIT/CD226, was described. TIGIT is a T-cell coinhibitory receptor that binds to CD155 on the dendritic cell surface, driving them to a more tolerogenic phenotype. Disturbance in this axis could be expected to contribute to development of autoimmune diseases [15].

The CD226 rs763361 variant is located in exon 7 encoding the cytoplasmic tail of CD226 which harbors two phosphorylation sites. The substitution from glycine to serine at residue 307 may affect phosphorylation at residues 322 and 329. Tyrosine phosphorylation at residue 322 of CD226 may be important for LFA-1 (*β*2 integrin Lymphocyte Function-Associated Antigen-1) mediated signaling in T cells. In addition, serine phosphorylation at residue 329 is also required for physical association of CD226 with LFA-1 in T cells. Alternatively, this SNP may alter RNA splicing by disrupting splice site enhancers or silencers, resulting in an isoform of CD226 with altered function [16, 17]

Besides TID, the nonsynonymous rs763361 variant has also been associated with several autoimmune diseases such as rheumatoid arthritis, celiac disease, Graves' disease, systemic lupus erythematosus, multiple sclerosis, systemic sclerosis, Wegener granulomatosis, and psoriasis mainly in Caucasian populations [18–21].

Reinforcing the role of CD226 in autoimmune disorders, a three-variant haplotype in* CD226*, rs763361-rs34794968-rs727088, was associated with systemic lupus erythematosus (SLE) and systemic sclerosis-related pulmonary fibrosis. However, it was proposed that in this context rs727088 could be the risk SNP interfering on CD226 transcription levels [22]. Indeed, the association of T1D and the rs763361 variant in* CD226* identified by GWAS could indicate that other variants might exist and contributed to this risk.

However, there was only a trend of possible association of rs763361 with SLE in Chinese and with T1D in Colombians [19] and there is no data about CD226 variants in patients with T1D in the very heterogeneous Brazilian population.

Therefore, the aim of this study was to investigate the contribution of CD226 sequence variants to susceptibility to type 1 diabetes in our population and to correlate risk variants with clinical data, autoantibody status, C peptide levels, and HLA (DR) alleles.

We found that rs763361 in* CD226* is associated with type 1 diabetes risk, mainly in females, and with greater frequency of GAD autoantibody and lower C-peptide levels, probably reflecting a more aggressive pattern of autoimmune aggression. We believe that this association in the very heterogeneous Brazilian population indicates an even stronger role for CD226 in susceptibility to T1D.

## 2. Materials and Methods

### 2.1. Subjects

532 T1D patients (mean age 23.9 ± 12.8 years, age at diagnosis 11.6 ± 7.5 years) were recruited from Hospital das Clínicas da Faculdade de Medicina da Universidade de Sao Paulo. All patients were diagnosed according to the criteria established by the American Diabetes Association (ADA) [1] and treated with two or more doses of insulin per day. The control group was composed by 592 health volunteers (28 ± 11.5 years) without family history of type 1 or 2 diabetes and autoimmune diseases and with normal fasting plasma HbA1c and glucose levels.

The study design was approved by Institutional Board Review and all subjects gave written informed consent.

### 2.2. Blood Parameters

Fasting plasma glucose was determined using an enzymatic colorimetric assay (LABTEST GOD-ANA, Brazil). HbA1c was measured using HPLC (CLAE) and fasting serum C-peptide levels by radioimmunoassay (HCP-20K, USA); the coefficients of variation (CV) intra- and inter-assay were, respectively, 4.5%–9.3%.

### 2.3. Polymerase Chain Reaction (PCR) and Sequencing Analysis

Genomic DNA was extracted from peripheral blood leukocytes by the salting-out procedure. The coding region (exons 2, 3, 4, 5, 6, and part of exon 7) of* CD226 *(ENSG00000150637) were amplified by polymerase chain reaction (PCR), using six primers pairs (Invitrogen Life Technologies, Brazil) in a Veriti Thermal Cycler (Applied Biosystems, Foster City, CA). PCR primers and conditions are available on request.

PCR amplified products were submitted to direct sequencing in an ABI 3130 capillary sequencer (Applied Biosystem, Foster City, CA) using the Big Dye terminator v3.1* cycle* sequencing Kit- (Applied Biosystem-Life Technologies) and analyzed with the Chromas Lite 2 and Sequencher 4.8 Programs.

### 2.4. *CD226 *Genotyping

Two SNPs (rs763361 and rs727088) were selected as genetic markers on the basis of literature reports. Genotyping was accomplished in 525 T1D patients and 592 controls using commercially available genotyping assays TaqMan (Applied Biosystems Life technologies, USA). All reactions were carried out using TaqMan Genotyping MasterMix (Applied Biosystems) on a StepOnePlus real-time PCR cycler (Applied Biosystems, USA) according to manufacturer's instructions. Genotyping was completed in >99% of the sample and the accuracy was >95%, determined by direct sequencing of 10% of all samples.

### 2.5. HLA (DR) Genotyping

HLA DRB1 alleles were amplified by PCR-SSP (Polymerase Chain Reaction-Sequence Specific Primers) using a generic kit of DRB1 diagnosing typing (Micro SSP* AlleleGeneric *HLA class II* DNA typing Tray-One-Lambda INC*).

### 2.6. Autoantibodies

Serum levels of autoantibodies against glutamic acid decarboxylase and tyrosine phosphatase were determined by radioimmunoassay (RSR limited, UK; CV < 7%). Normal values (NV) of 700 health controls (considering 3 SD) were >1.0 IU and >0.8 IU for anti-GAD and anti-IA2, respectively. The sensitivity of both assays is 0.2 IU. Autoantibodies against thyroid peroxidase (Auto Delfia, Turku, Finland, CV = 3.6%–8.2%; NV ≤ 35 U/mL), thyroglobulin (Auto Delfia, Turku, Finland; CV = 2.9%–5.7%; NV ≤ 35 U/mL), and 21-hydroxylase enzyme (RSR limited, UK; CV < 7%; NV > 1 IU/mL) were determined by radioimmunoassay.

Anti-nuclear (NV < 1/80), anti-liver/kidney microsomal type 1 antibody, anti-gastric parietal cell, anti-endomysium, and anti-smooth muscle (NV-negative) were quantified by indirect immunofluorescence. Rheumatoid factor (RF; NV < 20 IU/mL) was evaluated using nephelometry and TSH receptor autoantibody (RSR limited, UK; CV = 6%–10,6%; NV < 19%) by iodine radioreceptor assay.

### 2.7. Statistical Analysis

Statistical analyses were performed using the SPSS 13.0 software for windows. Allelic and genotypic association between* CD226* polymorphisms and T1D was carried out using Pearson's 2 × 2 contingency tables as implemented in Graph Pad 2.00 software. - Chi-square test was used to compare qualitative variables and the Fisher exact test for small samples. Numerical variables with parametric and nonparametric distributions were compared using Student's *t*-test and Mann-Whitney test, respectively. In the case of two samples comparison we used the nonparametric Wilcoxon test. Results were presented as means and standard deviations. For the analyses of effects of two independent variables on a qualitative dependent variable (presence or absence), we used a nominal logistic fit, available in the JMP 6.03 software (SAS Institute Inc., Cary, NC).

Genotype frequencies were tested for Hardy-Weinberg equilibrium (HWE) by chi-square test. The recessive model was used to analyze the genotypes of* CD226* allelic variants. *P* ≤ 0.05 was considered statistically significant. For the analysis of the effects of genetic markers, the critical *P* value was corrected by Bonferroni adjustments and indicated in the tables as *Pc*. Haplotype frequencies and pairwise linkage disequilibrium between variants were evaluated with the CubeX software (BGEL/CAiTE, University of Bristol, UK). Possible haplotypes for each individual were estimated using the Bayesian method in PHASE v2.1.1.

It was estimated (http://www.lee.dante.br/) that differences of allelic variant distribution could be detected with a power exceeding 80% (>95%) at the 0.05 level of significance.

## 3. Results 

### 3.1. Demographic Features of the Study Subjects

Demographic and laboratory data of subjects are provided in Table 1. As expected, HbA1c and glucose levels were higher and C-peptide levels were lower in patients (*P* < 0.01). HLA DR3 or -DR4 alleles prevailed in T1D patients compared to controls (*P* < 0.0001). European ancestry and females were more frequent in T1D patients as well as several autoantibodies. With the exception of Rheumatoid Factor, anti-21 hydroxilase, anti-liver/kidney microsomal type 1 antibody, and anti-smooth muscle antibody, all the other autoantibodies were significantly more frequent in T1AD patients than in the healthy controls (*P*_*c*_ < 0.003). The greater frequency of autoantibodies, in patients with T1D, was not related to gender (Table 2).

### 3.2. Frequency of Alleles, Genotypes, and Haplotypes of* CD226* Variants in T1D Patients and Controls

Twelve variants already described in public repositories (http://www.ensembl.org/) were detected by direct sequencing: three in exonic regions (rs72481820, rs72481819, and rs763361) and nine in exon-intron boundaries (rs75418532, rs57743896, rs1630425, rs73970293, rs77826708, rs1788101, rs3737395, rs62090790, and rs763362). No new variant was found.

Genotype frequencies were in Hardy-Weinberg Equilibrium in all variants for the control population. Allele, genotype, and estimated haplotype frequencies of variants in CD226 did not differ between T1D patients (*n* = 106) and controls (*n* = 102) (data not shown).

In sequence, two* CD226* variants that were previously related to autoimmune diseases were genotyped in 531 T1D patients and 590 controls. The rs763361 genotypes were found to be in HWE in the control group. The TT genotype prevailed in T1D group (33.5%) when compared with controls (25.7%) (OR = 1.45, 95% CI = 1.12–1.8, *P* = 0.0044) (Table 3). The second variant tested, rs727088, was found to be in strong linkage disequilibrium with rs763361 (*D*′ = −0.948, *χ* ^2^ = 453.08). The CC genotype was also associated with T1D patients susceptibility (31.4%* versus *24.6%; OR = 1.41; 95% CI = 1.08–1.84;   *P* = 0.0143) but, as the control group was not in Hardy-Weinberg equilibrium (*P* = 0.0186), it was excluded in further analyzes.

### 3.3. Effects of rs763361 TT Genotype on T1D Characteristics

The predisposing effect of rs763361 TT genotype to type 1 diabetes was independent of the ancestrality. It prevailed both in T1D patients of European ancestry (32.5% × 24.2%; OR = 1.51; 95% CI = 1.09–2.07, *P* = 0.013) as in those of non-European ancestry (41.2% × 28.0%; OR = 1.8; (95% CI = 1.09–2.98, *P* = 0.0281) when compared to controls. Compared to pooled CC + TC genotypes, the* CD226* rs763361 TT genotype had no association with high risk HLA genotypes (DR3 or DR4), which frequencies were similar between patients with TT (85.9%) or CT/CC genotypes (82.9%; *P* = 0.42), or with age at diagnosis (before or after 15-year old), *P* = 0.23.

Patients with new onset diabetes (less than 2 years of duration) carrying the rs763361 TT genotype also had lower serum levels of C peptide (0.4 ± 0.3 ng/dL) when compared with those with TC + CC genotypes (0.7 ± 0.5 ng/dL); *P* = 0.013.

When the frequencies of rs763361 TT genotype in T1D patients and controls were stratified for gender, the susceptibility risk was observed for T1D females (*P* = 0.0012), but not for males (Table 4).Further, when considering the entire population (patients with T1D and controls), those subjects carrying the rs763361 TT genotype had an increased frequency of anti-GAD65 autoantibody: 31.9% × 24.5%; OR = 1.57; CI = 1.136–2.194; and *P* = 0.0081 (Table 5). The mean titer of anti-GAD65 was higher in females with TT genotypes (9.8 ± 25.2 IU/mL) than in CC + CT females—at the CD226 rs763361—(4.3 ± 13.7, *P* = 0.0003). The anti-GAD mean titers for TT against CC + CT males were not significantly different (2.0 ± 8.2 IU/mL and 1.4 ± 6.0 IU/mL, resp.; *P* = 0.67), also in the pooled sample.

However, C peptide levels in recent onset patients with T1D were similar between TT females (0.42 ± 0.28 ng/mL) and males (0.39 ± 0.33); *P* = 0.79.

In addition, the rs763361 TT genotype of* CD226* had no effect on frequency of extrapancreatic autoantibodies related to other autoimmune diseases that usually coexist with T1D (Table 5).

## 4. Discussion 

In this report we show that rs763361 in exon 7 of* CD226* is associated with type 1 diabetes risk in a Brazilian Cohort (*P* = 0.0044). Our results replicate findings of previous European studies [10, 23] and were independent of ancestry. Despite of the great admixture of the Brazilian population, the T1D patients consisted mostly of Caucasians with HLA-DR3 or -DR4 HLA risk alleles [6]. Including, the direct sequencing of* CD226 *coding regions identified twelve variants previously described in other populations and no new variant was found.

New onset T1D patients (with less than two years of disease duration) bearing the rs763361 TT genotype presented lower C-peptide levels, indicating accelerated pancreatic beta-cell destruction and a more aggressive autoimmune phenomenon. Moreover subjects carrying the rs763361 TT genotype had an increased frequency of anti-GAD65 autoantibody. These novel findings might therefore reinforce* CD226*'s role in susceptibility to T1D and in disease features.

However, the real effect of this variant is still not completely understood. The amino acid exchange Gly307Ser attributed by the rs763361 within the CD226 protein could alter the two intracytoplasmic phosphorylation sites which are important in CD226 mediated signaling [16, 17]. As this protein is involved with cytotoxicity and differentiation of naïve T CD4+ cells any disturbance in these processes might contribute to exacerbation of the immune response. However, Wallace et al. [24] observed that T1D risk correlates with lower CD226 mRNA, which could reduce cell activation on cross-linking, altering the interactions of monocytes with lymphocytes and other immune cells and the CD226 receptors, CD112 and CD155.

The T1D risk associated with rs763361 was more evident in females than in males, determining increased anti-GAD titer and frequency, suggesting an additive effect of female hormones [25]. Gender is associated with differences in clinical presentation, onset, progression, and outcome of several autoimmune diseases, and females have stronger cellular and humoral immune reactions compared with males [26]. Elevated levels of estrogen and progesterone are supposed to induce Th2 responses, while low estrogen and prolactin concentrations have been reported to enhance Th1 responses. As a matter of fact, clinical evidences show that menstrual cycle, pregnancy, and menopausal status that are characterized by fluctuations of endogenous estrogens significantly influence the course of autoimmune diseases. In addition, estrogen treatment was found to protect isolated primary B cells from B cell receptor-mediated apoptosis. This fact suggested that estrogen could modulate survival and activation of B cells skewing the naïve immune system toward autoreactivity and proliferation [25]. C peptide levels in recent onset patients with T1D were similar between TT females (0.42 ± 0.28 ng/mL) and males (0.39 ± 0.33), *P* = 0.79, and probably reflect Th1 response.

Although the global incidence of T1D seems not to differ between male and female, countries with a high incidence of T1D have a high male to female ratio and those of low incidence have a low ratio [27]. The increased number of females in T1D group is consistent with findings of higher T1D prevalence among females in Brazil [9].

## 5. Conclusion 

We have found that rs763361 in* CD226* is associated with type 1 diabetes risk, greater frequency of GAD autoantibody, and lower C-peptide levels in a Brazilian cohort, reflecting a more aggressive pattern of autoimmune disease. We believe that this association in the very heterogeneous Brazilian population indicates an even stronger role for CD226 in susceptibility to T1D. The great risk conferred to female T1D patients is a recent find and deserves further analyses.

## Figures and Tables

**Table 1:** Demographic and laboratory characteristics of T1D patients and controls.

Characteristics	T1D	Controls	*P*
	Median ± SD or %	Median ± SD or %	
European ancestry	81.35%	61.75%	<0.0001
Gender female	59.4%	38.4%	<0.0001
Age (years)	23.92 ± 12.79	27.98 ± 11.56	<0.01
Glucose (mg/dL)	189.24 ± 115.54	83.2 ± 39.3	<0.01
HbA1c^a^ (%)	8.59 ± 2.19	5.32 ± 0.5	<0.01
C peptide (ng/dL)	0.35 ± 0.42	2.00 ± 1.41	<0.0001
HLA DR3 or DR4 alleles	84.2%	39.3%	<0.0001
Years of disease	12.2 ± 10.27	—	—
Autoantibodies positivity			
Anti-GAD65^b^	44.25%	1.4%	<0.0001
Anti-IA2^c^	42.4%	1.9%	<0.0001
Anti-TPO^d^	23.8%	12%	<0.0001
Anti-Tg^e^	23.6%	7.25%	<0.0001
ANA^f^	21.3%	3.8%	<0.0001
TRAb^g^	7.25%	0.37%	<0.0001
Anti-gastric Parietal cell	6.5%	0.5%	<0.0009
Anti-21 hydroxylase	5.3%	0.65%	0.0191
Anti-smooth muscle	3.5%	0.8%	0.0855
Rheumatoid factor	2.9%	1.0%	0.1801
Anti-LKM-1^h^	0.6%	0.4%	1.0000

^a^HbA1c: Glycated hemoglobin; ^b^AntiGAD65: glutamic acid decarboxylase antibody; ^c^Anti-IA2: tyrosine phosphatase antibody; ^d^Anti-TPO: anti- peroxidase antibody; ^e^Anti-Tg: anti-thyroglobulin antibody; ^f^ANA: anti-nuclear antibodies; ^g^TRAb: TSH receptor antibody; ^h^Anti-LKM-1: anti-liver/kidney microsomal type 1 antibody. *P* values were determined by chi-square test, Fisher's exact test, or Mann Whitney test, as appropriate. Critical *P*was corrected by Bonferroni method <0.002.

**Table 2:** Gender effects on frequency of autoantibodies in T1D patients and controls.

Characteristics	T1D^a^ patients		Controls		*P*
	Female	Male	Female	Male	
Anti-GAD65^b^	48.2%	38.0%	0.9%	1.6%	0.82
Anti-IA2^c^	40.2%	45.6%	1.8%	1.9%	0.66
Anti-TPO^d^	25.9%	20.2%	19.8%	9.0%	<0.005
Anti-Tg^e^	24.5%	23.2%	15.3%	4.0%	<0.005
ANA^f^	22.8%	18.5	25.0%	3.2%	<0.05
TRAb^g^	5.1%	7.9%	9%	0%	<0.05
Anti-gastric parietal cell	9.2%	1.5%	0%	0.4%	0.91
Anti-21 hydroxylase	5.7%	4.8%	0%	1.1%	0.37
Anti-smooth muscle	3.0%	3.1%	1.6%	0.3%	0.35
Rheumatoid factor	3.9%	1.3%	0%	1.1%	0.78
Anti-LKM-1^h^	0.9%	0%	0%	0.4%	1.00

^a^T1D: type 1 diabetes; ^b^AntiGAD65: glutamic acid decarboxylase antibody; ^c^Anti-IA2: tyrosine phosphatase antibody; ^d^Anti-TPO: anti- peroxidase antibody; ^e^Anti-Tg: anti-thyroglobulin antibody; ^f^ANA: anti-nuclear antibodies; ^g^TRAb: TSH receptor antibody; ^h^Anti-LKM-1: anti- liver/kidney microsomal type 1 antibody. The critical *P* values for the lines where the samples are separated by gender are for gender effect only in a nominal logistic fit. *P*was corrected by Bonferroni's criterium <0.0045.

**Table 3:** Genotype frequencies of *CD226* rs763361 and rs727088 variants in patients with T1D and control subjects.

Variant	T1D *n* (%)	Control *n* (%)	OR	CI	*P*
Genotype					
rs763361					
TT	178 (33.5)	152 (25.7)	1.453	1.12–1.80	**0.0054**
TC + CC	353 (66.5)	438 (74.3)			
rs727088					
CC	166 (31.4)	134 (24.6)	1.410	1.08–1.84	**0.0143**
CT + TT	362 (68.6)	412 (75.4)			

*P* values were determined by chi square test; OR: odds ratio; CI: confidence interval; critical *P* was corrected by the Bonferroni's method <0.025.

**Table 4:** Allele and genotype frequencies of *CD226* rs763361 variant in patients with type 1 diabetes and controls stratified by gender.

Variant rs763361	Gender	T1D *n* (%)	Controls *n* (%)	OR	CI	*P*
Genotype/allele						
TT	Female	111 (35.1)	50 (21.9)	1.928	1.30–2.84	**0.0012**
TC + CC		205 (64.9)	178 (78.1)			
T		360 (57.0)	227 (49.8)	1.335	1.04–1.70	**0.0224**
C		272 (43.0)	229 (50.2)			
TT	Male	67 (31.2)	102 (28.2)	1.154	0.79–1.66	0.504
TC + CC		148 (68.8)	260 (71.8)			
T		233 (54.2)	386 (53.3)	1.036	0.81–1.31	0.8213
C		197 (45.8)	338 (46.7)			

*P* values were determined by chi square test; OR: odds ratio; IC: confidence interval; critical *P*was corrected by the Bonferroni's method <0.025.

**Table 5:** Frequency of autoantibodies in pooled sample of patients and controls according to gender and *CD226* rs763361 genotypes.

Autoantibody	Females		*P* value	Males		*P* value
	TT	CT/CC		TT	CT/CC	
Anti-GAD^a^	35.9	21.7	0.0015	13.6	12.9	0.89
Anti-IA2^b^	28.8	18.8	0.02	13.1	16.2	0.42
Anti-Gastric Parietal cell	10.6	3.9	0.53	0.0	1.0	1.00
Anti-smooth muscle	3.9	3.5	1.00	0.9	1.3	1.00
Anti-LKM-1^c^	0.0	1.3	1.00	0.0	0.5	1.00
Anti-21-hidroxilase	4.8	2.8	0.62	2.3	2.7	1.00
ANA^d^	24.2	22.1	0.85	9.0	6.1	0.35
Rheumatoid Factor	0.0	6.0	0.08	0.0	1.7	0.58
Anti-TPO^e^	25.0	22.6	0.66	13.2	11.3	0.61
Anti-Tg^f^	23.2	18.3	0.36	10.5	8.4	0.56
TRAB^g^	5.9	5.1	1.00	3.2	0.9	0.14

^a^AntiGAD65: glutamic acid decarboxylase antibody; ^b^Anti-IA2: tyrosine phosphatase antibody; ^c^Anti-LKM-1: anti-liver/kidney microsomal type 1 antibody; ^d^ANA: anti-nuclear antibody; ^e^Anti-TPO: anti-peroxidase antibody; ^f^Anti-Tg: anti-thyroglobulin antibody; ^g^TRAb: TSH receptor antibody. *P* values were determined by the Fisher exact test; critical *P* was corrected by the Bonferroni's method <0.0045.
